# Influence of bleaching and desensitizing gel on bond strength of orthodontic brackets

**DOI:** 10.1590/2176-9451.20.2.049-054.oar

**Published:** 2015

**Authors:** Fernanda Alves Rodrigues Britto, Adriana Simoni Lucato, Heloisa Cristina Valdrighi, Sílvia Amélia Scudeler Vedovello

**Affiliations:** 1 Master's student in Orthodontics, Centro Universitário Hermínio Ometto (UNIARARAS), Araras, São Paulo, Brazil; 2 Professor, Centro Universitário Hermínio Ometto (UNIARARAS), Postgraduate program, Department of Orthodontics, Araras, São Paulo, Brazil

**Keywords:** Tooth bleaching, Hydrogen peroxide, Shear strength

## Abstract

**OBJECTIVE:**

The objective of this study was to assess, *in vitro,* the influence of bleaching gel and the use of desensitizing agent over bond strength of ceramic brackets bonded to bovine enamel.

**METHODS:**

One hundred bovine incisors were selected and randomly divided into five groups (n = 20): Group 1, control group (without bleaching); Group 2, bleached with 35% hydrogen peroxide; Group 3, bleached with 35% hydrogen peroxide (three applications, 15 minutes each) and desensitizing agent applied for 10 minutes; Group 4, bleached with 35% hydrogen peroxide for 40 minutes; Group 5, bleached with 35% hydrogen peroxide for 40 minutes with desensitizing agent applied for 10 minutes. Brackets were bonded 7 days after bleaching and submitted to shear bond strength test after 24 hours at a compression rate of 1 mm/minute. After fracture, the adhesive remnant index (ARI) was assessed under stereoscopic at 40 x magnification. Shear strength data (MPa) were submitted to one-way ANOVA and Tukey's test with significance level set at 5%.

**RESULTS:**

Group 5 (29.33 MPa) showed significantly higher bond strength than Group 1 (19.19 MPa), Group 2 (20.59 MPa) and Group 4 (23.25 MPa), but with no difference in comparison to Group 3. There was no significant difference among the other groups. The adhesive remnant index showed predominance of score 3, that is, all resin remained adhered to enamel for all groups.

**CONCLUSION:**

Bleaching with 35% hydrogen peroxide with calcium associated with desensitizing agent application produced higher bond strength values of brackets bonded to bovine enamel.

## Abstract (pt)

**OBJETIVO:**

o objetivo do presente estudo foi avaliar, *in vitro*, a influência do gel clareador e da utilização de dessensibilizante na resistência adesiva de braquetes cerâmicos colados ao esmalte bovino.

**MÉTODOS:**

cem incisivos bovinos foram aleatoriamente divididos em cinco grupos (n = 20). Grupo 1, sem clareamento (controle); Grupo 2, clareamento com peróxido de hidrogênio a 35%; Grupo 3, clareamento com peróxido de hidrogênio a 35%, sendo três aplicações de 15 minutos cada, e aplicação do dessensibilizante por 10 minutos; Grupo 4, clareamento com peróxido de hidrogênio a 35% durante 40 minutos; Grupo 5, clareamento com peróxido de hidrogênio a 35% durante 40 minutos e aplicação do dessensibilizante durante 10 minutos. Os braquetes foram colados sete dias após o clareamento e submetidos ao teste de resistência ao cisalhamento após 24 horas, a uma velocidade de compressão de 1mm/minuto. Após a fratura, avaliava-se o braquete, com lupa estereoscópica, com magnificação de 40x, e o Índice de Remanescente Adesivo (IRA). Os dados de resistência ao cisalhamento (MPa) foram submetidos à análise de variância e ao teste de Tukey, com nível de significância de 5%.

**RESULTADOS:**

os resultados mostraram que as amostras do Grupo 5 apresentaram resistência de união significativamente superior à dos grupos 1, 2 e 4, mas sem diferença do Grupo 3. Não houve diferença significativa entre a resistência de união dos demais grupos. O Índice de Remanescente Adesivo mostrou predominância do escore 3, ou seja, toda resina permaneceu aderida ao esmalte, para todos os grupos.

**CONCLUSÃO:**

pôde-se concluir que o clareamento com peróxido de hidrogênio a 35%, com cálcio associado à aplicação do dessensibilizante, produziu maior resistência dos braquetes ao esmalte bovino.

## INTRODUCTION

Esthetics is one of patients' demands when seeking dental offices to change the angle, position and color of their teeth.^1^ One of the most common esthetic complaints involves changes in the color of teeth, in addition to disproportionate shape and misalignment. Tooth bleaching is indicated to improve the esthetics of the smile, and must be performed before restorative and rehabilitative procedures.^2^

The most frequently used bleaching agents are hydrogen peroxide and carbamide peroxide in various concentrations, and bleaching may be performed by means of two techniques: at-home or in-office bleaching.^3^ In procedures performed at the dentist's office, 35% hydrogen peroxide is usually used, and should be applied and supervised by duly qualified professionals.^4^ Both techniques are equally effective.^5^

Tooth bleaching promotes rupture of the pigmented molecules impregnated in the dental structures, making them smaller, with significant reduction in the tonalities of their color, so that the tooth becomes whiter.^6^^,^ 7 ^,^ 8 Bleaching may be redone after 14 months.^8^

Various studies have demonstrated that changes occur in the morphology, microhardness and permeability of bleached enamel.^10^^,^ 11 This is believed to occur due to the demineralizing potential of bleaching agents.^11^ Thus, some undesirable effects may be generated.

One of the undesirable effects reported by patients is tooth sensitivity which occurs due to an increase in enamel and dentin permeability by the bleaching agents.^11^ This allows penetration of fluids into the dentinal tubules, stimulating nerve fibers and consequently leading to sensitivity. This may be reverted by the use of desensitizing agents, with emphasis on the use of potassium nitrate, which reduces sensitivity by means of diminishing the ability of nerve fibers in dental pulp to transmit pain;^12^ and fluorides that obstruct dentinal tubules and, thereby, also promote the inhibition of pain.^13^^,^ 14

Adding calcium to the composition of bleaching agents is another alternative to reduce the adverse effects caused by bleaching. The presence of calcium in the composition of bleaching agents probably contributes, directly and indirectly, to reduce sensitivity.^15^ The goal of this addition is to increase the saturation of gels with ions, thereby reducing mineral losses and increasing enamel resistance to demineralization caused by peroxides.^16^ Studies have evinced that the presence of calcium in 35% hydrogen peroxide-based bleaching agents increased microhardness of bleached enamel, thus resulting in remineralization of this substrate.^17^ Therefore, the addition of calcium and fluoride helps to control mineral loss in enamel submitted to different bleaching treatments.^18^

Studies have reported increase in resin bond strength to enamel submitted to bleaching.^19^^,^ 20 The application of bleaching agents may also cause surface porosities that change enamel permeability and interfere in bond strength in bracket bonding.^21^ There is some discrepancy in the results found in the literature with respect to the reduction in bond strength of brackets bonded to bleached teeth. However, changes caused by bleaching are undeniable, making it necessary to wait for a period of at least seven days in order to perform orthodontic bracket bonding more safely.^22^

Patients who need orthodontic treatment and choose esthetic brackets give much importance to the smile. Some of them have stained teeth and request that bleaching treatment be performed prior to orthodontic treatment. This is because they do not want to remain with stained teeth throughout all orthodontic treatment, which can last more than three years. Therefore, it is important to assess the influence of bleaching treatment associated with desensitizing agents on the bond strength of brackets to enamel.

Therefore, the aim of this study was to assess the influence of bleaching agents, with and without calcium, and desensitizing agents on the bond strength of ceramic brackets bonded to bovine enamel, in addition to assessing the adhesive remnant index (ARI) after the bond strength test.

## MATERIAL AND METHODS

This study began after being approved by UNIARARAS Institutional Review Board under protocol #560/2011.

One hundred recently extracted bovine teeth were selected, based on the following inclusion criteria: teeth with intact crowns, absence of white spot lesions, cracks and fractures, and teeth originating from the same lot. These teeth were included in PVC tubes with polystyrene (Resina Cristal, Piraglass, Piracicaba, SP, Brazil), with the buccal surface exposed, and submitted to prophylaxis with pumice stone and water, using a Robson brush. Teeth were divided into five groups (n = 20), according to the bleaching and desensitizing agent.

» Group 1: control group in which specimens received no bleaching before bracket bonding.

» Group 2: bleached with 35% hydrogen peroxide (Whiteness HP Maxx 35%, FGM, Joinville, Santa Catarina, Brazil), with three applications for 15 minutes each, as recommended by the manufacturer, using the light polymerizing appliance Radii (SDI, Australia). At the end of treatment, the samples were washed with water and polished with a felt disc.

» Group 3: bleached with 35% hydrogen peroxide (Whiteness HP Maxx 35%, FGM, Joinville, Santa Catarina, Brazil) according to the protocol described for Group 2. After bleaching, teeth were washed and dried, and the desensitizing agent was applied (KF 2%, FGM, Joinville, Santa Catarina, Brazil) for 10 minutes, followed by washing and polishing with a felt disc.

» Group 4: bleached with 35% hydrogen peroxide (Whiteness Blue 35% Calcium, FGM, Joinville, Santa Catarina, Brazil) for 40 minutes, as recommended by the manufacturer, and subsequently washed, dried and polished with a felt disc.

» Group 5: bleached with 35% hydrogen peroxide (Whiteness Blue 35% Calcium, FGM, Joinville, Santa Catarina, Brazil) for 40 minutes. After bleaching, teeth were washed and dried, and the desensitizing agent was applied (KF 2%, FGM, Joinville, Santa Catarina, Brazil) for 10 minutes, followed by washing and polishing with a felt disc.

After the bleaching procedure, all samples were stored in distilled water for seven days after which the brackets were bonded. Prophylaxis of the bonding area was performed with pumice stone and a Robson brush. The enamel was etched with 37% phosphoric acid for 15 seconds and subsequently washed and dried. Afterwards, Transbond XT Primer (3M Unitek, Monrovia, USA) adhesive was applied and light activated for 10 seconds with the Radii (SDI, Australia) light curing appliance. Transbond XT (3M Unitek, Monrovia, USA) resin was applied at the base of the Edgewise prescription ceramic brackets for maxillary central incisors (Morelli, Sorocaba, SP, Brazil). Brackets were manually positioned on the tooth surface until the material flowed, and were then polymerized for 40 seconds, 10 seconds on each surface (distal, mesial, gingival and occlusal).

Specimens were stored in distilled water at 37 ^o^C for 24 hours^23^^,^ 24 and submitted to shear bond strength test in an Instron 4411 universal testing machine (Instron Corp, USA) at a compression speed rate of 1 mm/minute. Shear bond strength was tested for normal distribution by means of Kolmogorov-Smirnov test. One-way ANOVA and Tukey's multiple comparison tests were used to assess the influence of bleaching on shear bond strength results at a significance level of 5%.

After the shear bond strength test, the adhesive remnant index (ARI) was assessed in accordance with the method proposed by Artun and Bergland,^25^ and determined by scores that ranged from 0 to 3:

" Score 0 - absence of any residue of adhesive layer on enamel;
" Score 1 - presence of less than half resin remnant on enamel;
" Score 2 - presence of more than half resin remnant on enamel;
" Score 3 - presence of all resin remnant on enamel, together with impression of the bracket base design.

Adhesive remnant was evaluated under a stereoscopic microscope (Carl Zeiss, MC 63A, Germany) under 40 x magnification**.**

## RESULTS

Analysis of variance showed significant difference among the different types of material (P < 0.001). Tukey's test (Table 2) showed that the samples submitted to bleaching with HP Blue and later application of 2% KF desensitizing agent presented significantly higher bond strength in comparison to control and the samples submitted to bleaching with HP Blue and HP Maxx; however, no differences were found in comparison to those bleached with HP Maxx and later application of 2% KF desensitizing agent. There was no significant difference between the bond strength values of other groups.

**Table 1 -:** Study groups divided according to the bleaching agent and desensitizing gel.

Group	Bleaching agent	Desensitizing
Group 1 (control)	___	___
Group 2	35% hydrogen peroxide (Whiteness HP Maxx, FGM)	___
Group 3	35% hydrogen peroxide (Whiteness HP Maxx, FGM)	Desensitizing (KF 2%, FGM)
Group 4	35% hydrogen peroxide (Whiteness Blue Calcium, FGM)	___
Group 5	35% hydrogen peroxide (Whiteness Blue Calcium, FGM)	Desensitizing (KF 2%, FGM)

**Table 2 -:** Shear strength (MPa) of brackets bonded to enamel and subjected to different bleaching protocols.

Material	Shear strength (MPa)
Group 5: HP Blue + KF a 2%	29.33 (6.03) ^A^
Group 4: HP Blue	23.25 (6.85) ^B^
Group 3: HP Maxx + KF a 2%	24.22 (5.45) ^AB^
Group 2: HP Maxx	20.59 (7.17) ^B^
Group 1: Control	19.19 (6.12) ^B^

Different letters stand for significant difference (P < 0.05).

**Table 3 -:** Frequency (%) of ARI distribution after applying different bleaching agents.

Scores	HP Blue + 2% KF	HP Blue	HP Maxx + 2% KF	HP Maxx	Control
Score 0	1 (5%)	1 (5%)	1 (5%)	2 (10%)	1 (5%)
Score 1	0 (0%)	0 (0%)	0 (0%)	0 (0%)	2 (10%)
Score 2	1 (5%)	2 (10%)	0 (0%)	2 (10%)	4 (20%)
Score 3	18 (90%)	17 (85%)	19 (95%)	16 (80%)	13 (65%)

**Figure 1 -:**
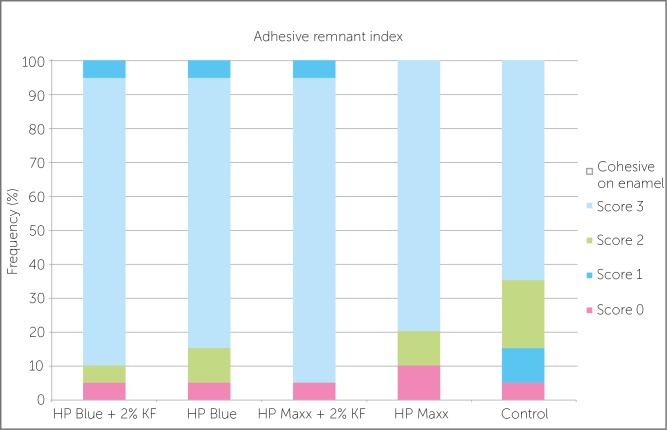
Frequency (%) of adhesive remnant index after application of different bleaching agents.

Assessment of the adhesive remnant index (ARI) showed predominance of score 3; that is, all resin remained adhered to the enamel for all groups. For the control group, there was a trend towards scores 1 and 2 (Fig 1). The groups bleached with HP Blue, with and without desensitizing agent, and the HP Maxx group with desensitizing agent presented cohesive fracture in enamel.

## DISCUSSION

Many patients have some trouble using orthodontic appliances, even ceramic ones. In order to reduce the color contrast between teeth and ceramic brackets, patients are subjected to bleaching procedures before bracket bonding, which leads orthodontists to question the influence of bleaching agent on the bonding procedure.

One of the most common effects resulting of hydrogen peroxide or carbamide peroxide on bleaching treatment in Dentistry is the change in bond strength to enamel and dentin. The reduction in bond strength of resin to enamel and dentin may be related to the presence of free radicals of oxygen that interfere in the polymerization of resin material.^26^^,^ 27 Therefore, the most used clinical approach to eliminate or reduce the effects of residual oxygen is to wait a few days after the end of bleaching treatment before bonding brackets.^28^^,^ 29

This study demonstrated that the samples submitted to bleaching with HP Blue Calcium and subsequent application of 2% KF desensitizing agent presented a significantly higher bond strength compared with non-bleached teeth and teeth bleached without later desensitizing application. Assessment of adhesive remnant index (ARI) showed predominance of score 3, failures between bracket and resin, thereby confirming that bleaching did not negatively influence the bond strength of brackets to enamel.

The high bond strength values shown in this study are possibly due to the time interval of seven days between the end of bleaching and orthodontic bracket bonding. Within this period, residual oxygen must have been neutralized, thus not interfering in polymerization of resin composite for bonding. This may be proved by the absence of significant differences between the bleached groups and the control group (without bleaching). Some authors^30^^,^ 31 suggest that orthodontic bonding should be delayed for one week after bleaching so as to ensure adequate bond strength. Other authors^32^^,^ 33 also recommend postponing bonding after bleaching for periods ranging from 24 hours to four weeks. Thus, according to these authors,^22^^,^ 34 a period of at least seven days of waiting time is necessary so that orthodontic bracket bonding can be performed more safely. This is in agreement with the results obtained in the present study in which the waiting time was of seven days before bonding orthodontic brackets to bovine enamel after bleaching.

The release of oxygen may cause morphological alterations in mineralized tissues.^4^^,^ 8 ^,^ 33 The reduction in bond strength of dentin to bleached teeth has been related to changes in the mineral and protein content of enamel, and not to the effect of residual oxygen.^35^ Soares *et al* 36 demonstrated that the addition of calcium and fluoride ions to the gel reduced mineral loss, thereby increasing resistance to demineralization.

Giannini *et al* 16 reported that if mineral ions were added to the gel during bleaching and ionic exchange, they could perhaps be taken up and increase enamel resistance to demineralization. Since fluoride and calcium ions increase the saturation of the bleaching gel, lower mineral loss could occur during bleaching; therefore, gels with the addition of fluoride or calcium could reduce or overcome the adverse effects of bleaching treatment. This result was obtained in the present study, in which the presence of calcium in the bleaching gel and application of a desensitizing agent must have remineralized this substrate, showing an increase in bond strength in teeth bleached with a bleaching agent containing calcium, and application of the desensitizer.

Another result found in this study was that the association of bleaching agent with calcium and desensitizing agent produced the highest shear bond strength. Nevertheless, a previous study found that bleaching with the application of desensitizing agent significantly reduced the bond strength of orthodontic brackets bonded to human enamel.^37^ Reduction in bond strength in that study was probably due to a post-bleaching bonding time of two days, in disagreement with studies that indicate that the ideal should be waiting for at least seven days, as it was done in the present experiment. This waiting time would be necessary for complete release of residual oxygen.^29^ Moreover, the calcium present in the bleaching agent could reduce demineralization during tooth bleaching, and the fluoride ions released from desensitizing agent could promote ions change with enamel and form fluoridated apatite on tooth surface that could be related to the highest shear bond strength for Group 5.

Another study reported that in order to reduce the effects of residual oxygen on bonding procedures performed right after bleaching, fluoride in gel could be applied to the enamel.^27^ The presence of sodium fluoride in the desensitizing agent may act as a remineralizing agent, thereby forming a layer of calcium fluoride on the enamel surface.^38^ Thus, the application of the desensitizing agent may have removed the layer of residual oxygen, which did not interfere negatively in orthodontic bracket bonding to bleached enamel.

Although the ideal substrate for this type of study is the human tooth, bovine teeth were used as a substitute because extracted human teeth are becoming difficult to obtain due to progress in conservative dental treatment.^38^ Bovine teeth are easily obtainable and are reported to be a reliable substitute for human teeth in enamel bonding.^39^

Clinically, the application of desensitizing agent after bleaching with hydrogen peroxide can be a good option to reduce the negative effects of residual oxygen on bond strength of brackets bonded to bleached enamel. However, further studies must be conducted to investigate the effect of associating bleaching gel with calcium and desensitizing agent applied to the dental structure.

## CONCLUSION

Bleaching agent with calcium associated with desensitizing gel increased the shear bond strength of ceramic brackets bonded to the enamel. Bleaching did not reduce shear strength between bracket and teeth.

The adhesive remnant index (ARI) revealed that score 3 was predominant, that is, all resin remained adhered to the enamel.
